# The Psychometric Properties of an Internet-Administered Version of the Depression Anxiety and Stress Scales (DASS) in a Sample of Dutch Adults

**DOI:** 10.1007/s10862-017-9626-6

**Published:** 2017-09-18

**Authors:** Klaas J. Wardenaar, Rob B. K. Wanders, Bertus F. Jeronimus, Peter de Jonge

**Affiliations:** 1 Department of Psychiatry, Interdisciplinary Center Psychopathology and Emotion Regulation (ICPE), University of Groningen, University Medical Center Groningen (UMCG), P.O. box 30.001, 9700RB Groningen, The Netherlands; 2 Faculty of Behavioural and Social Sciences, Department of Developmental Psychology, University of Groningen, Groningen, The Netherlands

**Keywords:** DASS, Psychometric, Validation, Item response theory, Internet-based, Linking

## Abstract

Psychometric work on the widely used Depression Anxiety and Stress Scales (DASS) has mostly used classical psychometrics and ignored common internet-administered versions. Therefore, the present study used not only classical, but also modern psychometrics based on item response theory (IRT) to evaluate an internet-administered version of the DASS (Dutch translation). Internet-administered DASS data were collected as part of a large internet-based study in the Dutch adult population (*n* = 7972). Initially, external correlates (i.e. demographics other measures) and some classical psychometrics (internal consistency, convergent/divergent validity) of the DASS scales were evaluated. Next, IRT was used to investigate the scales’ dimensionality, discrimination and item-functioning. Finally, the DASS depression scale was further investigated by linking it to the more clinically-oriented Quick Inventory of Depressive Symptomatology (QIDS) using item response theory (IRT). Initial classical psychometric analyses supported the scales’ internal consistency (alpha = 0.94–0.98) and convergent/divergent validity. IRT analyses showed that each of the DASS scales was only suitable to measure variations in a very narrow and rather mild severity range. Linking the DASS depression scale with the QIDS also showed that the DASS depression scale discriminated best in the mild-moderate severity range, but not at higher severity levels that were covered by the QIDS. In conclusion, the scales of the internet-administered DASS show good internal consistency and validity. However, users should be aware that the scales discriminate best at mild-moderate severity ranges in the general population.

## Introduction

The Depression Anxiety and Stress Scales (DASS) is a 42-item self-report instrument that was developed to improve the discrimination between depression and anxiety (Lovibond and Lovibond 1995a, b). The DASS is widely used and several psychometric studies have shown the internal consistency, convergent/divergent validity and factorial structure to be satisfactory (Lovibond and Lovibond 1995a; Brown et al. 1997; Page et al. 2007). Despite abovementioned work, the dimensionality, discriminatory ability and item-functioning of the DASS remain incompletely understood. The previous work has only employed classical psychometric analyses that provide limited information about these important measurement characteristics (e.g., Sijtsma 2009). Fortunately, these aspects can be effectively investigated with modern psychometric methods based on item response theory (IRT; Embretson and Reise 2000). In addition to providing information about the functioning of scales and items within an instrument, IRT allows for deeper investigations of the relationships between scores on one measurement scale with scores on another scale. IRT-based linking, for instance, can be used to map scores on two separate scales that are designed to measure similar constructs (e.g., depression) onto a common underlying severity dimension (Kolen and Brennan 2004; Orlando et al. 2000; Wahl et al. 2014). This mapping provides valuable information about two scales’ relatedness in terms of measurement range and discriminative properties, and can be used to evaluate if a measure has the properties needed for administration in specific target groups. Unfortunately, IRT work has only been conducted with the shorter DASS-21 (Shea et al. 2009; Parkitny et al. 2012) but, to our knowledge, not with either a paper-and-pencil or internet-administered version of the full-length DASS.

Another subject that has received relatively little attention in the literature is the psychometric quality and measurement characteristics of the DASS when administered via the internet. Some classical psychometric work that was conducted with an internet-administered version of the full-length DASS (Zlomke 2009), showed that the scales had good internal consistency (alpha = 0.93–0.95). However, modern psychometric (i.e. Rasch) analyses have only been conducted with the internet-administered DASS-21 (Shea et al. 2009). An extensive IRT-based study of the full-length internet-administered DASS could give more insight into the potential usefulness and added value of the instrument for large-scale and low-cost mental health research (e.g., Coles et al. 2007; Naglieri et al. 2004; Gosling and Mason 2015). Several advantages of online mental health assessments are: (a) the lower rates of socially desirable responding and decreased social anxiety (e.g., Joinson 1999), (b) the possibility to include those otherwise unable or unwilling to visit a research site (internet samples tend to be substantially more diverse than conventional samples), and (c) the potential for using (computerized) adaptive testing to shorten assessment time and personalize measurements (Gibbons et al. 2008; Buchanan 2002; Gosling and Mason 2015). Ideally, the psychometric characteristics of internet-administered versions of instruments should be investigated with dedicated studies, as findings for paper-and-pencil versions of questionnaires do not necessarily generalize to internet-administered versions (Buchanan 2002).

The current study addresses the above described issues by evaluating the classical and modern psychometric properties of an internet-administered Dutch version of the DASS in a group of population-dwelling Dutch adults (*N* = 7972). First, preliminary classical psychometric analyses were conducted (internal consistency and convergent/divergent validity). Next, IRT was used to investigate each scale’s measurement properties (i.e. discriminative ability; range of measurement). To gain more insight into the meaning and functioning of the DASS depression scale in the context of more broadly-defined clinical depression, IRT-based linking was used to place the DASS depression scores onto a common scale with scores on the Quick Inventory of Depressive Symptomatology (QIDS) that is conceptually closer to the clinical definition of major depressive disorder (MDD; Diagnostic and Statistical Manual fifth edition) and includes a broader set of clinically relevant criterion symptoms (i.e. several somatic/vegetative symptoms and suicidality). These analyses were used to gain some insight into the extent to which the DASS depression-scale scores actually capture severity variations in clinically-defined depression severity.

## Method

### Participants and Procedures

The data were collected as part of a large scale project (van der Krieke et al. 2016), which was aimed to investigate the distribution of mental health dimensions in the Dutch population, focusing both on mental vulnerabilities/problems (e.g., mood, anxiety stress) and mental strengths (e.g., positive affect, humor, empathy, well-being). The project was advertised through a press release by the University Medical Center Groningen (UMCG), after which it was picked up by local and national media. Participants could go to the project website (www.hoegekis.nl), create an account, and fill in a questionnaire assessing basic socio-demographics (e.g., age, gender education, social status, employment). After this, participants could choose to complete different modules of questionnaires (e.g., affect/mood, well-being, mental strengths; van der Krieke et al. 2016). After completion of each module, participants received automated feedback about their scores, including a comparison with the other participants’ scores. Participants were informed about the project and the fact that their data were to be stored, anonymized and used for scientific research before deciding to continue and participate in the research project. The study protocol was reviewed by the Medical Ethical Committee of the UMCG and exempted because it concerned a nonrandomized open study targeted at anonymous volunteers in the general public. In a period of exactly one year (December 13th 2013 – December 13th 2014), 12,501 subjects registered online with the research project. Of these, 7972 (63.8%) completed the DASS. Those who did complete the DASS were older (mean: 46.2 years [s.d. = 14.9] vs. 43.7 years [s.d. = 14.9]; *t* = −9.2, *p* < 0.001; Cohen’s D = 0.17) and more often female (67.5% female vs. 61.1% male; χ^2^ = 52.7, p < 0.001; Cramer’s V = 0.07) compared to those who did not fill in the DASS (*n* = 4529; 36.2%). All questionnaires were administered via the internet and participants could only submit their responses if all items in a questionnaire were completed.

### Measures

The DASS (Lovibond and Lovibond 1995a, b; Dutch translation: de Beurs et al. 2001) is a 42-item self-report questionnaire with items rated on a 4-point (0–3) Likert scale. The DASS consists of three subscales of 14 items to assess the specific emotional dimensions, viz., ‘depression’, ‘anxiety’, and ‘stress’. The Dutch translation of the DASS was previously found to have good psychometric properties (De Beurs et al. 2001).

The official Dutch translation of the Quick Inventory of Depressive Symptomatology self-report (QIDS; Rush et al. 2003) was used to measure depression severity (translation details are provided on the IDS website: www.ids-qids.org). The QIDS is a questionnaire consisting of 16 items rated on a 4-point Likert scale (0–3) and covers all criterion symptoms of a depressive episode according to the DSM. Nine scores are counted up to a total depression severity score (range: 0–27). Only the highest score on the sleep items (1–4), appetite and weight loss/gain items (items 6–9) and the highest score on the psychomotor agitation/retardation items (items 15–16) are used in the sum score. The paper-and-pencil version of the QIDS was previously shown to have good internal consistency (alpha = 0.86; Rush et al. 2003), convergent validity and construct validity (Reilly et al. 2015). Studies specifically investigating the Dutch QIDS translation found adequate psychometric properties (e.g., Lako et al. 2014).

The Dutch translation of the Positive Affect and Negative Affect Schedule (PANAS; Watson et al. 1988; Dutch translation: Peeters et al. 1996) is a self-report questionnaire consisting of 20 items rated on a 5-point Likert scale (1–5) that assess the presence of several emotions in the week prior to the assessment including today (in the used version). The PANAS consists of two 10-item subscales: Negative Affect (NA) covers negative emotions and distress (e.g., feeling ‘guilty’, ‘pessimistic’) and Positive Affect (PA) covers positive emotions (e.g., feeling ‘interested’, ‘enthusiastic’). In the current study, an internet-administered version of the PANAS was administered. Previously, the PANAS has been shown to have good internal consistency (alpha = 0.84–0.89 for NA and alpha = 0.84–0.89 for PA; Watson et al. 1988; Crawford and Henry 2004). The Dutch translation has also been shown to have good psychometric properties (Peeters et al. 1996; Engelen et al. 2006).

### Statistical Analyses

Associations between DASS scales and sociodemographic factors were investigated by comparison of median DASS scores between sociodemographic groups using non-parametric Mann-Whitney U tests (for comparison of 2 groups) and Kruskal-Wallis (K-W) tests (for comparison of 3 groups). Effect-sizes for these non-parametric analyses were calculated using the formulas presented in Fritz et al. (2012) and Cohen (2014). These analyses were conducted with R (version 3.4.0; R Core Team 2015).

### Classical Psychometrics

Cronbach’s alpha and average inter-item correlations were calculated based on the polychoric item-correlation matrix. Spearman correlation coefficients were calculated to investigate the inter-relationships between each of the DASS scales, and of the DASS scales with the QIDS and the PANAS scales. These analyses were conducted with R-package ‘psych’ (version 1.7.5; Revelle 2015).

### Item Response Theory

Prior to IRT analyses, each scale’s unidimensionality was checked by running a 3-factor exploratory factor analysis (EFA) with a bifactor rotation, and inspecting the proportion of explained variance for the first extracted factor, using ≥70% explained variance as the cutoff for sufficient unidimensionality (Reise et al. 2010). If sufficiently unidimensional, each scale was investigated with IRT analyses. Because the DASS items had an ordinal response scale, a Graded Response Model (GRM; Samejima 1969) was fitted to each of the DASS subscales. This model estimates two parameters for each item: the discrimination parameter (α) describes how strongly an item is related to the underlying severity dimension, and the threshold parameter (β) describes the severity of the symptom described by the item. In a constrained model, α is constrained to be the same across items, which implies that all items are equally strongly related to the underlying dimension, similar to a polytomous Rasch model. In an unconstrained model, α is estimated for each item separately. Both model-variants were fitted and compared with a likelihood ratio test. For the best-fitting model, the items’ thresholds and discrimination parameters were inspected. In addition, the scales’ test information curves (TIC) were inspected to gain insight into the information each scale provided along the underlying severity dimension. The item information curves (IIC) were also inspected to evaluate specific items’ individual contributions to measurement information. IRT analyses were conducted with R-package ‘ltm’ (version 1.0-1; Rizopoulos 2006).

### Linking

The items of the DASS depression scale and the QIDS were mapped on a common scale to investigate how measurements with the DASS depression scale were related to measurements with the QIDS, in terms of discriminative ability and the severity range of measurement. To facilitate this *linking* process, a set of constants (based on similar items in both scales) was identified for calibration. The following item-pairs were identified based on similarities in content: (a) feeling sad/depressed (DASS-13 and QIDS-5; polychoric correlation (*r*_pch_) = 0.86); (b) loss of interest (DASS-16 and QIDS-13; *r*_pch_ = 0.74); and (c) feelings of worthlessness in comparison to others (DASS-17 and QIDS-11; *r*_pch_ = 0.81). For the linking analyses, item parameters for the DASS depression scale and QIDS were estimated first with a GRM in ltm. Next, the items of both instruments were placed on a common scale. To do this, the IRT-parameters of the QIDS items (thresholds and discrimination) were rescaled to the scale of the DASS depression scale, which was used as reference. This was done by use of linking constants obtained with the Stocking-Lord calibration linking method (Stocking and Lord 1983). Linking analyses were rerun with an alternative calibration method (Haebara 1980) to investigate the consistency of the results. Next, item-parameters of both instruments were investigated and the IICs were inspected and compared between the items of the two questionnaires, in order to gain insight into their comparative coverage along the depression severity spectrum. Finally observed DASS scores were equated to observed QIDS scores (original scoring), based on corresponding theta-values on the shared underlying depression severity dimension. Linking was performed with the R-package ‘plink’ (version 1.5-1; Weeks 2010).

## Results

### Sample Description

The majority of participants was female (*n* = 5382; 67.5%) and the mean age was 46.2 years (s.d. = 12.4). Most participants were employed (74.1%), were married or in a steady relationship (73.8%), and had a college education (78.3%). The median QIDS score (5.0; IQR: 2.0–8.0) indicated absent to mild depression according to published norms (Rush et al. 2003). According to the Rush et al. (2003) cut-offs, 27.8% had mild (6–10), 10.9% had moderate (11–15) and 4.8% had severe or very severe (16+) depression severity. The median scale scores on the internet-administered DASS depression scale (4.0; interquartile range [IQR]: 1.0–10.0), anxiety scale (2.0; IQR: 0.0–5.0) and stress scale (7.0; IQR: 3.0–12.0) indicated normal symptom levels according to published norms by Lovibond and Lovibond (1995a, b). According to the DASS cut-off scores by Lovibond and Lovibond (1995a, b), 9.3% had mild (score: 10–13), 9.6% had moderate (14–20), and 7.2% had severe or extremely severe (21+) depression levels. In addition, 4.2% had mild (8–9), 6.4% had moderate (10–14), and 4.6% had severe or extremely severe (15+) anxiety levels and 8.4% had mild (15–18), 6.8% had moderate (19–25), and 2.7% had severe or extremely severe (26+) stress levels. For subgroup-specific analyses, gender-groups were formed, and three age-groups were distinguished based on the tertiles of the age-distribution (18–39 years [*n* = 2616], 40–54 years [*n* = 2669], 55–87 years [*n* = 2684]). Gender and age-groups were cross-tabbed to construct gender-by-age subgroups.

Median DASS scale scores were significantly higher in females, in young age-groups, in the unmarried group, in the unemployed group, and in those with less than a college education (Table 1), although the observed effect sizes were small.

**Table 1:** Median DASS scale scores and their interquartile ranges in different sociodemographic groups

		DASS scales		
		Depression	Anxiety	Stress
Gender	Female:	4.0 (1.0–10.0)	2.0 (1.0–5.0)	8.0 (4.0–13.0)
	Male:	3.0 (1.0–9.0)	1.0 (0.0–4.0)	6.0 (2.0–11.0)
		Z = −4.58 p < 0.001 η^2^ = 0.003	Z = −8.74 *p* < 0.001; η^2^ = 0.010	Z = −10.3 K-W: p < 0.001 η^2^ = 0.013
Age group^a^	Age: 18–39:	4.0 (1.0–11.0)	2.0 (1.0–6.0)	8.0 (4.0–14.0)
	Age: 40–54:	4.0 (1.0–10.0)	2.0 (0.0–4.0)	7.0 (3.0–12.0)
	Age: 55–87:	3.0 (1.0–10.0)	2.0 (0.0–4.0)	6.0 (3.0–11.0)
		H = 24.2 p < 0.001 η^2^ = 0.003	H = 102.5 p < 0.001 η^2^ = 0.013	H = 139.0 p < 0.001 η^2^ = 0.017
Partner	Unmarried/no partner:	6.0 (2.0–14.0)	3.0 (1.0–6.0)	8.0 (3.0–13.0)
	Married/steady partner:	3.0 (1.0–9.0)	2.0 (0.0–4.0)	7.0 (3.0–12.0)
		Z = −14.5 p < 0.001 η^2^==0.026	Z = −10.3 p < 0.001 η^2^==0.013	Z = −4.2 p < 0.001 η^2^ = 0.002
Work	Unemployed:	5.0 (2.0–14.0)	3.0 (1.0–7.0)	8.0 (3.0–14.0)
	Employed:	3.0 (1.0–9.0)	2.0 (0.0–4.0)	7.0 (3.0–12.0)
		Z = −13.0 p < 0.001 η^2^ = 0.021	Z = −12.6 p < 0.001 η^2^ = 0.020	Z = −6.0 p < 0.001 η^2^ = 0.005
Education	Less than college:	6.0 (2.0–14.0)	3.0 (1.0–7.0)	9.0 (4.0–15.0)
	College education:	3.0 (1.0–9.0)	2.0 (0.0–4.0)	7.0 (3.0–12.0)
		Z = −11.9 p < 0.001 η^2^ = 0.018	Z = −11.5 p < 0.001 η^2^ = 0.017	Z = −9.5 p < 0.001 η^2^ = 0.011

For the Kruskal Wallis test with three groups (k = 3), η^2^ is calculated as: H- k + 1/n-k (Cohen 2014)

For the Mann Whitney U test, η^2^ is calculated as Z^2^/n (Fritz et al. 2012)

Z = test statistic of the Mann-Whitney U test

H = test statistic of the Kruskal-Wallis test

^a^Based on tertiles of the age distribution

### Classical Psychometric Characteristics

Cronbach’s alpha coefficients (see Appendix Table 5) indicated very high internal consistency for each of the scales (alpha = 0.94–0.98). Average inter-item correlations were high for the anxiety (0.55), stress (0.56) and depression (0.74) scales. In addition, the DASS scales showed strong inter-correlations (ρ = 0.60–0.69). Spearman correlations between the DASS scales and the QIDS (alpha = 0.88), NA (alpha = 0.91) and PA (alpha = 0.93) indicated moderate to strong interrelatedness, with the strongest correlations being observed between the DASS depression scale and the QIDS (ρ = 0.77), between the DASS stress scale and NA (ρ = 0.74) and between the DASS depression scale and PA (ρ = −0.68). The weakest correlation was observed between DASS anxiety and PA (ρ = −0.43). The results were stable across gender, age-groups, and gender-by-age subgroups.

### Item Response Theory Analyses

Bifactor EFAs of the individual scales showed that the first general factor explained more than 70% of the variance in each of the DASS scales indicating sufficient unidimensionality for IRT analyses. For each of the DASS scales the unconstrained GRM fit the data better than the constrained GRM (Depression: LRT = 2517.1; df = 13, *p* < 0.01; Anxiety: LRT = 1923.3; df = 13, p < 0.01; Stress: LRT = 2368.4; df = 13, p < 0.01). This indicated that items differed with respect to their discriminatory ability.

### Depression Scale

**Fig. 1:**
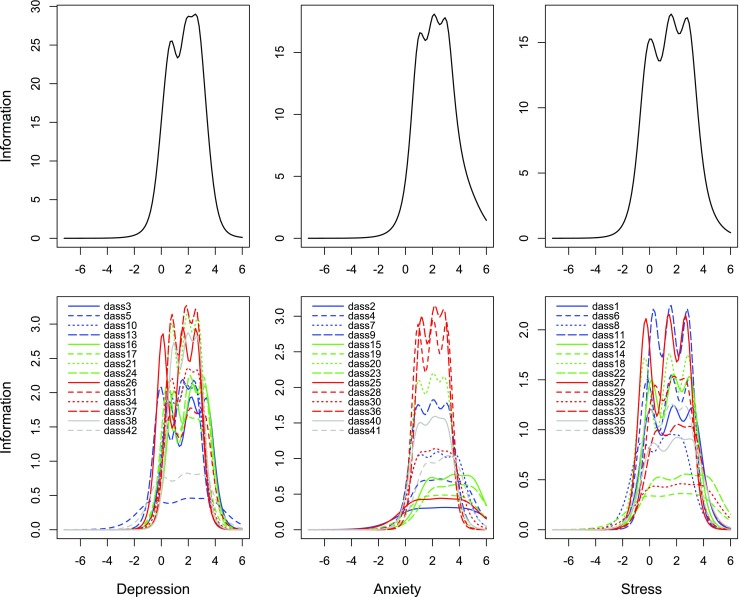
Test information curves (TIC) and item information curves (IIC) for the depression, anxiety and stress scales of the DASS in the total sample (n = 7972)

The lower end of the depression scale (see Table 2) was covered by symptoms of mood and motivational disturbance, e.g., ‘sad/depressed mood’ (item 13), ‘feeling down’ (item 26), ‘difficulty to get going’ (item 5). The highest end of the measured severity dimension was covered by items tapping into anhedonia, such as ‘lack of enthusiasm’ (item 31), ‘no positive feelings’ (item 3), and ‘no interest in anything’ (item 16). The TIC and IICs (Fig. 1) indicated that items varied in terms of the amount and severity range of the provided information. Several items provided remarkably high levels of information, for example item 37 (‘I could see nothing in the future to be hopeful about’) and item 21 (‘I felt that life wasn’t worthwhile’), which provided 18.1% of the total information. Contrarily, item 5 (‘I just couldn’t seem to get going’) and item 42 (‘I found it difficult to work up the initiative to do things‘) were relatively uninformative about severity and provided only 7% of the total information. An examination of the items’ thresholds and discrimination parameters showed several subsets of items with comparable measurement properties. For instance, items 17 (‘I felt I wasn’t worth much as a person’), 21 (‘I felt that life wasn't worthwhile), 34 (‘I felt I was pretty worthless’), 37 (‘I felt there was nothing to look forward to’), and 38 (‘I felt that life was meaningless’) showed strongly overlapping thresholds, in line with their overlapping content. This was also observed for items 24 (‘I couldn't seem to get any enjoyment out of the things I did’) and 31 (‘I was unable to become enthusiastic about anything’).

**Table 2:** Item response theory parameters for the items of the Depression, Anxiety and Stress scales of the DASS

	DASS items		Thr1	Thr2	Thr3	Mean Threshold	Discr
Depression scale	13	I felt sad and depressed	−0.07	1.49	2.51	1.31	2.87
	26	I felt down-hearted and blue	0.08	1.56	2.60	1.41	3.38
	5	I just couldn’t seem to get going	−0.65	1.83	3.65	1.61	1.28
	42	I found it difficult to work up the initiative to do things	−0.07	1.73	3.17	1.61	1.72
	10	I felt that I had nothing to look forward to	0.55	1.73	2.66	1.65	2.79
	17	I felt I wasn’t worth much as a person	0.48	1.82	2.82	1.70	2.44
	37	I could see nothing in the future to be hopeful about	0.75	1.79	2.64	1.73	3.50
	34	I felt I was pretty worthless	0.70	1.89	2.76	1.78	2.94
	21	I felt that life wasn’t worthwhile	0.74	1.88	2.76	1.79	3.44
	38	I felt that life was meaningless	0.88	1.92	2.75	1.85	3.25
	24	I couldn’t seem to get any enjoyment out of the things I did	0.47	2.08	3.18	1.91	2.93
	31	I was unable to become enthusiastic about anything	0.65	2.10	3.18	1.98	2.57
	3	I couldn’t seem to experience any positive feeling at all	0.57	2.17	3.38	2.04	2.72
	16	I felt that I had lost interest in just about everything	0.91	2.28	3.31	2.16	2.82
Anxiety scale	20	I felt scared without any good reason	0.92	2.03	3.00	1.98	2.84
	28	I felt I was close to panic	0.91	2.05	3.08	2.01	3.36
	9	Situations made me so anxious I was most relieved when they ended	0.86	2.04	3.14	2.02	2.59
	40	I was worried about situations in which I might panic and make a fool of myself	0.94	2.08	3.05	2.02	2.37
	36	I felt terrified	1.14	2.13	2.93	2.07	3.41
	30	I feared that I would be “thrown” by some trivial but unfamiliar task	1.01	2.21	3.39	2.20	2.00
	7	I had a feeling of shakiness	0.98	2.39	3.80	2.39	2.00
	25	I was aware of the action of my heart in the absence of physical exertion	0.70	2.56	4.33	2.53	1.24
	2	I was aware of dryness of my mouth	0.63	2.73	4.67	2.68	1.03
	4	I experienced breathing difficulty	1.13	2.65	4.28	2.69	1.59
	41	I experienced trembling	1.49	2.85	3.74	2.69	1.87
	19	I perspired noticeably in the absence of high temperatures or physical exertion	1.20	2.80	4.39	2.80	1.29
	15	I had a feeling of faintness	1.78	3.49	4.93	3.40	1.66
	23	I had difficulty in swallowing	2.06	3.66	5.02	3.58	1.48
Stress scale	8	I found it difficult to relax	−0.62	1.16	2.38	0.97	1.84
	27	I found that I was very irritable	−0.30	1.39	2.69	1.26	2.90
	18	I felt that I was rather touchy	−0.37	1.44	2.86	1.31	2.62
	39	I found myself getting agitated	−0.05	1.51	2.79	1.41	2.16
	6	I tended to over-react to situations	−0.20	1.58	3.03	1.47	2.44
	11	I found myself getting upset rather easily	0.27	1.54	2.82	1.54	2.94
	29	I found it hard to calm down after something upset me	0.33	1.70	2.76	1.59	2.36
	12	I felt that I was using a lot of nervous energy	0.11	1.72	3.03	1.62	2.43
	1	I found myself getting upset by quite trivial things	0.01	1.74	3.23	1.66	2.17
	35	I was intolerant of anything that kept me from getting on with what I was doing	0.17	1.90	3.26	1.78	1.83
	14	I found myself getting impatient when I was delayed in any way	−0.34	1.96	3.79	1.80	1.12
	33	I was in a state of nervous tension	0.47	1.98	3.25	1.90	1.95
	32	I found it difficult to tolerate interruptions to what I was doing	0.10	2.19	4.01	2.10	1.27
	22	I found it hard to wind down	0.53	2.58	4.32	2.47	1.41

Per scale, items are ordered in ascending order by mean threshold

Thr1 = response threshold between category 0 and 1; Thr2 = threshold between response category 2 and 3; Thr3 = threshold between response category 3 and 4; Discr = item discrimination parameter

### Anxiety Scale

The lower end of the anxiety severity dimension was covered by items assessing anxiety and panic, such as ‘feeling scared’ (item 20), ‘feeling close to panic’ (item 28), and ‘situational anxiety’ (item 9). The highest end of the anxiety dimension was covered by items assessing somatic arousal symptoms, such as ‘perspiration’ (item 19), ‘feeling faint’ (item 15), and ‘difficulties in swallowing’ (item 23). Inspection of Fig. 1 showed that item 28 (‘I felt I was close to panic‘) and 36 (‘I felt terrified‘) provided most information. The curves of items 2 (‘I was aware of dryness of my mouth’), 19 (‘I perspired noticeably’) and 25 (‘I was aware of the action of my heart’) showed that they provided little information along the dimension (apart from some information at the severe end). Several items contributed most of their information at the severe end of the dimension: item 15 (‘I had a feeling of faintness’) and item 23 (‘difficulty swallowing’). Inspection of the item-parameters indicated that there was some overlap in item-functioning in the anxiety scale, with the clearest overlap between items 9 (‘I found myself in situations that made me so anxious I was most relieved when they ended’), 20 (‘I felt scared without any good reason’), 28 (‘I felt I was close to panic’) and 40 (‘I was worried about situations in which I might panic and make a fool of myself’).

### Stress Scale

The low end of the stress scale was covered by items that assess symptoms of agitation and irritability, such as ‘difficulties to relax’ (item 8), ‘feeling very irritable’ (item 27), and ‘feeling touchy’ (item 18). The severe end was marked by items covering symptoms like ‘nervous tension’ (item 33), ‘intolerance to interruptions’ (item 32), and ‘difficulties to wind down’ (item 22). Inspection of Fig. 1 showed that individual items differed substantially in terms of the amount of information they provided along the severity dimension. Items 11 (‘I found myself getting upset rather easily’) and 27 (‘I found that I was very irritable’) provided high levels of information, whereas items 14 (‘I found myself getting impatient when I was delayed in any way’), 22 (‘I found it hard to wind down’) and 32 (‘I found it difficult to tolerate interruptions’) provided relatively little information along most of the dimension. However, only the latter items provided any information at the severe end. In the stress scale, overlap between items’ functioning was less pronounced than in the other scales.

### Linking DASS Depression and the QIDS

**Fig. 2:**
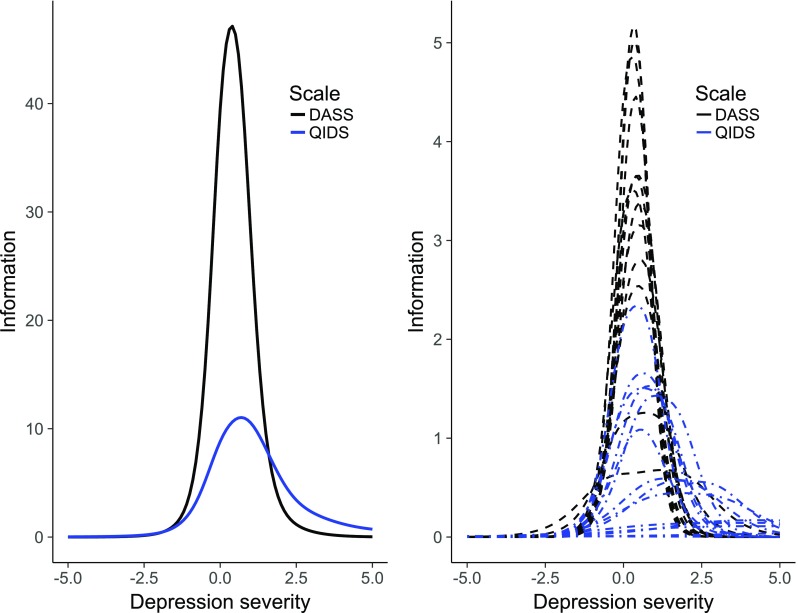
Test information curves (left) and item information curves (right) for the DASS depression scale (black lines) and the QIDS (blue lines) on the joint underlying depression severity dimension

The item-parameters of the DASS depression scale items and the QIDS items, ordered by increasing mean threshold on the common underlying scale are shown in Table 3. The two items at the extreme ends of the spectrum showed very low discriminative ability, and were therefore not included in the interpretation of the results. For the remaining items, the range of covered severity was large (lowest threshold at −0.66 and highest threshold at 4.74). The DASS items showed average thresholds of 1.02 to 1.75, and thresholds ranging from −0.66 to 3.01 and the QIDS items showed average thresholds ranging from 1.16 to 3.34 (thresholds ranging from: −0.01 to 4.74). This indicates that the DASS items were more located in the lower-middle range of the common severity spectrum, which was also evident from the IICs in Fig. 2. Among the DASS items, only two often-endorsed QIDS items were located at the mild end of the spectrum (QIDS11: ‘view of myself’, and QIDS5: ‘feeling sad’). Most QIDS items provided measurement information in the middle-high range of the common severity dimension. Among these items were DSM criterion symptoms for depression, not included in the DASS depression scale (i.e., appetite/weight change and psychomotor problems). Similar results were found with another linking method (Haebara), and when using the DASS instead of the QIDS as reference scale (see Appendix Tables 6, 7, 8 and 9). Equation of DASS depression to equivalent QIDS scores (original scoring) is shown in Table 4.

**Table 3:** DASS depression items are rescaled to the QIDS scale using the Stocking Lord linking calibration method. The items are ordered by increasing average threshold

Item	Label	Mean Thr	Thr 1	Thr2	Thr3	Discr
QIDS2^a^	Sleep during the night	0.67	−5.07	−1.64	8.71	0.21
DASS13	I felt sad and depressed	1.02	−0.16	1.17	2.04	3.36
DASS26	I felt down-hearted and blue	1.10	−0.04	1.23	2.12	3.95
QIDS11	View of myself	1.16	0.71	1.35	1.43	1.95
QIDS5	Feeling sad	1.17	−0.01	1.35	2.16	2.75
DASS5	I just couldn’t seem to get going	1.27	−0.66	1.46	3.01	1.50
DASS42	I found it difficult to work up the initiative to do things	1.27	−0.16	1.38	2.60	2.02
DASS10	I felt that I had nothing to look forward to	1.30	0.37	1.37	2.17	3.26
DASS17	I felt I wasn’t worth much as a person	1.35	0.30	1.45	2.30	2.86
DASS37	I could see nothing in the future to be hopeful about	1.37	0.53	1.43	2.16	4.09
DASS34	I felt I was pretty worthless	1.42	0.49	1.51	2.26	3.43
QIDS10	Concentration/decision making	1.42	0.31	1.44	2.52	2.30
DASS21	I felt that life wasn’t worthwhile	1.43	0.53	1.50	2.25	4.02
QIDS14	Energy loss	1.43	0.14	1.37	2.79	2.20
DASS38	I felt that life was meaningless	1.48	0.65	1.53	2.25	3.80
DASS24	I couldn’t seem to get any enjoyment out of the things I did	1.52	0.29	1.67	2.61	3.43
DASS31	I was unable to become enthusiastic about anything	1.59	0.45	1.69	2.61	3.00
DASS3	I couldn’t seem to experience any positive feeling at all	1.64	0.38	1.75	2.79	3.18
DASS16	I felt that I had lost interest in just about everything	1.75	0.67	1.84	2.72	3.29
QIDS13	General Interest	1.82	0.81	2.03	2.64	2.22
QIDS16	Feeling restless	2.33	1.09	2.16	3.75	1.20
QIDS15	Feeling slowed down	2.33	1.18	2.14	3.68	2.15
QIDS12	Thoughts of death/suicide	2.36	1.13	1.90	4.04	1.40
QIDS6_7	Appetite change	2.42	1.59	2.53	3.12	1.36
QIDS1	Falling asleep	2.70	0.92	2.76	4.40	0.69
QIDS3	Waking up too early	2.75	1.28	2.55	4.41	0.60
QIDS8_9	Weight change	3.34	2.24	3.03	4.74	0.75
QIDS4^a^	Sleeping too much	8.20	2.51	8.50	13.60	0.40

Items are ordered in ascending order by mean threshold. Thr1 = response threshold between category 0 and 1; Thr2 = threshold between response category 2 and 3; Thr3 = threshold between response category 3 and 4; Discr = item discrimination parameter. The 4 QIDS sleep items, 2 psychomotor problem items, and weight change and appetite change were not merged for these analyses

^a^These items showed very low discrimination parameters and were not included in the interpretation of the results

**Table 4:** Comparing observed DASS depression scores and estimated QIDS scores

Observed scores		
DASS depression score	QIDS total^a^	Theta
1–5	1	−2.90- -1.44
6–11	2	−1.24- -0.46
12–15	3–6	−0.29 – 0.39
16–20	7–11	0.62–1.25
21–25	12–17	1.38–1.91
26–30	18–21	2.05–2.61
31–35	22–24	2.74–3.29
36–40	25–26	3.45–4.36
40+	27	4.83 +

Score equation based on Stocking-Lord linking, placing DASS scores on the original QIDS scale

^a^Equated QIDS scores rounded to nearest integer and based on Stocking-Lord linking, placing DASS depression scores on the QIDS score, using the original scoring (see Appendix Tables 6, 7, 8 and 9): sets of sleep items (1–4), weight and appetite items (6–9), psychomotor items (15 & 16) each combined into a single item score

## Discussion

This paper presented an investigation of the psychometric properties of an internet-administered version of the DASS in a sample of Dutch adults. Previous work showed high internal consistency for the DASS scales, while associations with other instruments indicated good convergent/divergent validity, especially for the depression scale. In line with these previous findings, the current results show that the scales of the internet-administered version also have good classical psychometric properties. Additional modern psychometric analyses showed that the items within each DASS scale showed varying severity and discrimination parameters, although some overlap in item-functioning was observed in the depression and anxiety scales. The measurement information provided by items along the underlying severity dimension also varied within each scale and showed most variation in the anxiety and stress scales. Linking the DASS depression scale items to the items of the QIDS showed that, within the context of a more heterogeneous, clinically defined depression severity spectrum, the DASS items mostly measure in the mild-moderate range of depression severity.

The high alpha coefficients (0.94–0.98) indicated very good internal consistency for the DASS scales. However, together with the high average inter-item correlations (0.55–0.74), these coefficients also suggested that the DASS scales were quite homogeneous in their coverage, especially the DASS depression scale. This is probably because this scale includes overlapping items that measure quite narrow concepts (i.e. depressive cognitions and mood) resulting in a scale that measures a narrow construct (Clark and Watson 1995). Indeed, another direct comparison of the DASS-21 depression scale and the QIDS in a clinical sample showed higher internal consistency for the DASS-21 depression scale, which the authors explained by the fact that the DASS-21 scale is rather homogeneous (mainly cognitive and emotional symptoms) compared to the more comprehensive QIDS, which covers all clinical criteria for a major depressive disorder, including sleeping problems, appetite/weight change, energy-loss and psychomotor retardation/agitation (Weiss et al. 2015). Indeed, deeper investigation of the depression scale with IRT analyses showed strong overlap in item-functioning between items with similar content. For instance, sets of items that all assessed cognitions of worthlessness (items 17, 21, 34, 37 and 38) and items that all assessed lack of positive emotions (items 24 and 31) showed strong overlap. From a theoretical perspective, the fact that many items function in the same way, implies that the severity dimension as indexed by the complete scale score has a restricted range. Clusters of similarly functioning items provide a lot of information about a rather small severity interval. Indeed, when mapped on a common severity scale, the DASS-items provided most measurement information at the lower end of the overall depression severity spectrum, whereas typical criterion symptoms of clinical depressive episodes that are included in the QIDS but not in the DASS depression scale (i.e. psychomotor symptoms, appetite/weight change and hypo/hypersomnia) were endorsed at higher severity levels. Importantly, this indicates that the DASS depression scale cannot provide meaningful information along the whole spectrum of depression severity, which could result in ceiling-effects when the scale is used in more severely depressed populations.

Note that it is not negligence that the DASS included items that are rather similar in content, as the original authors aimed to divide each scale into even more specific ‘subscales’ of 2–5 items (Lovibond and Lovibond 1995a, b). For instance, the depression scale was meant to assess the following domains: ‘dysphoria’, ‘hopelessness’, ‘devaluation of life’, ‘self-deprecation’, ‘lack of interest/involvement’, ‘anhedonia’ and ‘inertia’. However, our results suggest that items of self-deprecation (item 21), devaluation of life (item 38) and hopelessness (item 37) functioned very similarly, indicative of a limited differentiation between these subdomains.

As stated above, the results show that the DASS depression scale is most useful to differentiate between mild-moderate severity levels. The finding of potentially redundant items may suggest that the depression scale, and possibly the other scales as well, can be shortened without compromising their differentiating ability within this range. Indeed, the short DASS-21 (Lovibond and Lovibond 1995b) includes only seven items per scale and has been quite thoroughly investigated using classical (e.g. Antony et al. 1998; Clara et al. 2001; Sinclair et al. 2012; Osman et al. 2012; Gomez et al. 2014) and modern (Shea et al. 2009; Parkitny et al. 2012) psychometric techniques. However, the depression scale of the DASS-21 still includes sets of items that were found to overlap in this study (DASS-21 items 17 and 21 [worthlessness/meaninglessness] and items 3 and 16 [lack of positive feelings/enthusiasm]). Based on the present findings, further shortening of the DASS scales could be considered. For instance, calculations in the current dataset showed that shortening the DASS depression scale to 5 items would still result in a scale with good internal consistency (alpha = 0.92; with DASS-21 item 5 [‘I found it difficult to work up the initiative to do things’] and item 21 [‘I felt that life was meaningless’] removed). Although this observation was based on data collected with the full-length DASS, it is in line with previous Rasch analyses (Shea et al. 2009), which suggested that the depression scale could be improved by removing item 5 (‘I found it difficult to work up the initiative to do things’). Alternatively, the DASS depression scale could be extended with a range of more diverse symptoms (e.g. vegetative symptoms) to increase the heterogeneity of the covered domains and the scale’s measurement range.

Although the properties of the anxiety scale could not be investigated in as much detail because secondary measures of anxiety were not administered, its average inter-item correlation was considerably lower (0.55) than for the depression scale. Although this indicates that scale homogeneity was less marked, some overlap in item functioning was observed in the IRT results, with four items that cover ‘situational anxiety’ (items 9 and 40) and ‘subjective experiences of anxious affect’ (items 20 and 28) providing most of their measurement information at the same severity level. Additionally, information at the mild-moderate end of the anxiety spectrum was mostly provided by items covering situational and subjective anxiety (i.e. panic, feeling scared), whereas information on the moderate-severe end of the spectrum was provided by items covering symptoms of autonomic/somatic arousal (i.e. trembling, perspiring, difficulty swallowing).

Within the stress scale, the average inter-item correlation was also lower than for the depression scale (0.56), but was still high enough to indicate some item redundancy. Although inspection of the IRT parameters of the stress scale showed that there were no sets or clusters of items with strongly overlapping functioning, most items were located relatively close together on the latent dimension (as indicated by their averaged item thresholds). This suggests that there is also room for improvement for the stress scale.

The current study had several strengths, including the large sample size, which provided the possibility to investigate the DASS’s psychometric properties in different demographic groups. Additional strengths were the use of modern psychometric techniques, and the linking of DASS depression scores with scores on the QIDS. However, some study limitations should be kept in mind. First, the data were collected in volunteers through an internet-platform, which attracted respondents that were relatively highly educated and often female. Consequently, the generalizability of the results to the general population - or subpopulations that are not covered by the current study - requires further investigation. Second, the full version of the DASS was used, instead of the shorter and often used DASS-21. The generalizability of the psychometric performance results from the current study to the short-form version needs further evaluation. Third, for the DASS anxiety and stress scales convergent validity could not be investigated very deeply, because more specialized anxiety and stress measures were not administered. Consequently the linking analyses could only be performed for the DASS depression scale. Finally, the sample was recruited from the general population and no information was available about formal (DSM-5) anxiety/depressive disorder diagnoses, limiting possibilities to test the scales’ relationships with diagnosed clinical psychopathology.

A promising direction for further research in the context of online-administered depression and anxiety instruments - including the DASS, is the implementation of computerized adaptive testing. The current results already provide some insight into how the scales’ items are distributed along their respective underlying severity spectra (Wahl et al. 2014). Such information is a good starting point for the development of algorithms that can quickly and effectively zero in on a person’s severity level, by strategically adapting each next administered item to the responses given on the previous items. Such algorithms could save administration time and would make measurement more personal (e.g., less administration of items that do not apply to the respondents) while increasing precision.

In conclusion, the present classical and modern psychometric investigation showed the internet-administered version of the DASS to (a) have good classical psychometric properties, (b) contain sets of items with similar item-functioning, and (c) be most suitable to measure dimensional depression severity variations in population samples (mild-moderate severity levels).

## Appendix 1

**Table 5:** The Depression Anxiety and Stress Scales (DASS) internal consistency coefficients, scale correlations and correlations with other measures in the total sample and in gender-groups, age-groups and gender-by-age subgroups

Sample	scales	All ages			Age-groups^a^								
					18–39 years (*n* = 2619)			40–54 years (n = 2669)			55–87 years (n = 2684)		
		D	A	S	D	A	S	D	A	S	D	A	S
Total sample (n = 7972)	D	0.98	–	–	0.98	–	–	0.98	–	–	0.98	–	• *–*
	A	0.60	0.94	–	0.61	0.95	–	0.60	0.95	–	0.61	0.94	–
	S	0.69	0.65	0.95	0.69	0.67	0.94	0.70	0.65	0.95	0.69	0.64	0.95
	QIDS	0.77	0.61	0.67	0.78	0.61	0.68	0.77	0.61	0.68	0.74	0.61	0.64
	NA	0.66	0.63	0.74	0.66	0.63	0.73	0.67	0.62	0.76	0.67	0.62	0.72
	PA	−0.68	−0.43	−0.49	−0.67	−0.42	−0.48	−0.69	−0.42	−0.50	−0.69	−0.44	−0.48
Male sample (*n* = 2590)^b^	D	0.98	–	–	0.98	–	–	0.98	–	–	0.98	–	–
	A	0.61	0.95	–	0.59	0.95	–	0.61	0.95	–	0.62	0.93	–
	S	0.69	0.63	0.95	0.65	0.59	0.94	0.70	0.65	0.95	0.71	0.64	0.95
	QIDS	0.74	0.58	0.63	0.76	0.56	0.63	0.75	0.60	0.64	0.73	0.59	0.63
	NA	0.68	0.61	0.73	0.68	0.60	0.68	0.66	0.60	0.77	0.69	0.62	0.73
	PA	−0.68	−0.43	−0.46	−0.67	−0.42	−0.41	−0.69	−0.42	−0.46	−0.68	−0.45	−0.49
Female Sample (n = 5382)^c^	D	0.98	–	–	0.98	–	–	0.98	–	–	0.98	–	–
	A	0.60	0.94	–	0.62	0.94	–	0.59	0.94	–	0.59	0.94	–
	S	0.69	0.66	0.95	0.70	0.68	0.94	0.70	0.64	0.95	0.68	0.64	0.95
	QIDS	0.78	0.61	0.68	0.79	0.62	0.70	0.79	0.60	0.68	0.75	0.61	0.64
	NA	0.65	0.63	0.74	0.65	0.64	0.74	0.67	0.62	0.75	0.65	0.61	0.71
	PA	−0.68	−0.43	−0.50	−0.67	−0.42	−0.50	−0.69	−0.43	−0.51	−0.69	−0.43	−0.48

All Spearman’s ρ correlation coefficients; underlined Cronbach’s alpha coefficients are printed on the diagonals; alphas were calculated using polychoric item matrices; D = depression scale; A = anxiety scale; S = stress scale; QIDS = Quick Inventory of Depressive Symptomatology; NA = Negative Affect; PA = Positive Affect

^a^Based on tertiles of the age distribution

^b^Sizes of age subgroups: 18–39 years: *n* = 631; 40–54 years: *n* = 789; 55–87 years: *n* = 1160

^c^Sizes of age subgroups: 18–39 years: *n* = 1978; 40–54 years: *n* = 1880; 55–87 years: *n* = 1524

## Appendix 2: Additional linking analyses

**Table 6:** DASS depression items are rescaled to the QIDS scale using 3 calibration items and the Haebara calibration method

item	label	Thr 1	Thr2	Thr3	Mean Thr	Discr
QIDS2	Sleep during the night	−5.07	−1.64	8.71	0.67	0.21
DASS13	I felt sad and depressed	0.08	1.24	2.01	1.11	3.84
QIDS11	View of myself	0.71	1.35	1.43	1.16	1.95
QIDS5	Feeling sad	−0.01	1.35	2.16	1.17	2.75
DASS26	I felt down-hearted and blue	0.19	1.29	2.07	1.18	4.52
DASS5	I just couldn’t seem to get going	−0.36	1.50	2.85	1.33	1.71
DASS42	I found it difficult to work up the initiative to do things	0.08	1.42	2.50	1.33	2.30
DASS10	I felt that I had nothing to look forward to	0.54	1.42	2.12	1.36	3.73
DASS17	I felt I wasn’t worth much as a person	0.48	1.49	2.23	1.40	3.26
DASS37	I could see nothing in the future to be hopeful about	0.69	1.47	2.10	1.42	4.68
QIDS10	Concentration/decision making	0.31	1.44	2.52	1.42	2.30
QIDS14	Energy loss	0.14	1.37	2.79	1.43	2.20
DASS34	I felt I was pretty worthless	0.65	1.54	2.19	1.46	3.92
DASS21	I felt that life wasn’t worthwhile	0.68	1.53	2.19	1.47	4.60
DASS38	I felt that life was meaningless	0.78	1.56	2.19	1.51	4.34
DASS24	I couldn’t seem to get any enjoyment out of the things I did	0.48	1.68	2.50	1.55	3.92
DASS31	I was unable to become enthusiastic about anything	0.61	1.70	2.50	1.61	3.43
DASS3	I couldn’t seem to experience any positive feeling at all	0.55	1.75	2.66	1.65	3.64
DASS16	I felt that I had lost interest in just about everything	0.81	1.83	2.60	1.75	3.76
QIDS13	General Interest	0.81	2.03	2.64	1.82	2.22
QIDS16	Feeling restless	1.09	2.16	3.75	2.33	1.20
QIDS15	Feeling slowed down	1.18	2.14	3.68	2.33	2.15
QIDS12	Thoughts of death/suicide	1.13	1.90	4.04	2.36	1.40
QIDS6_7	Appetite change	1.59	2.53	3.12	2.42	1.36
QIDS1	Falling asleep	0.92	2.76	4.40	2.70	0.69
QIDS3	Waking up too early	1.28	2.55	4.41	2.75	0.60
QIDS8_9	Weight change	2.24	3.03	4.74	3.34	0.75
QIDS4	Sleeping too much	2.51	8.50	13.60	8.20	0.40

**Table 7:** QIDS items are rescaled to the DASS depression scale using 3 calibration items and the Stocking Lord calibration method

item	label	Thr 1	Thr2	Thr3	Mean Thr	Discr
QIDS2	Sleep during the night	−5.81	−1.80	10.32	0.90	0.18
DASS13	I felt sad and depressed	−0.07	1.49	2.51	1.31	2.87
DASS26	I felt down-hearted and blue	0.08	1.56	2.60	1.41	3.38
QIDS11	View of myself	0.95	1.70	1.79	1.48	1.67
QIDS5	Feeling sad	0.11	1.70	2.66	1.49	2.34
DASS5	I just couldn’t seem to get going	−0.65	1.83	3.65	1.61	1.28
DASS42	I found it difficult to work up the initiative to do things	−0.07	1.73	3.17	1.61	1.72
DASS10	I felt that I had nothing to look forward to	0.55	1.73	2.66	1.65	2.79
DASS17	I felt I wasn’t worth much as a person	0.48	1.82	2.82	1.70	2.44
DASS37	I could see nothing in the future to be hopeful about	0.75	1.79	2.64	1.73	3.50
DASS34	I felt I was pretty worthless	0.70	1.89	2.76	1.78	2.94
QIDS10	Concentration/decision making	0.49	1.80	3.07	1.79	1.97
DASS21	I felt that life wasn’t worthwhile	0.74	1.88	2.76	1.79	3.44
QIDS14	Energy loss	0.29	1.72	3.39	1.80	1.88
DASS38	I felt that life was meaningless	0.88	1.92	2.75	1.85	3.25
DASS24	I couldn’t seem to get any enjoyment out of the things I did	0.47	2.08	3.18	1.91	2.93
DASS31	I was unable to become enthusiastic about anything	0.65	2.10	3.18	1.98	2.57
DASS3	I couldn’t seem to experience any positive feeling at all	0.57	2.17	3.38	2.04	2.72
DASS16	I felt that I had lost interest in just about everything	0.91	2.28	3.31	2.16	2.82
QIDS13	General Interest	1.06	2.50	3.21	2.26	1.90
QIDS16	Feeling restless	1.40	2.65	4.51	2.85	1.02
QIDS15	Feeling slowed down	1.51	2.63	4.44	2.86	1.83
QIDS12	Thoughts of death/suicide	1.44	2.35	4.86	2.88	1.20
QIDS6_7	Appetite change	1.99	3.09	3.78	2.95	1.16
QIDS1	Falling asleep	1.20	3.36	5.28	3.28	0.59
QIDS3	Waking up too early	1.62	3.11	5.28	3.34	0.51
QIDS8_9	Weight change	2.74	3.67	5.68	4.03	0.64
QIDS4	Sleeping too much	3.07	10.08	16.05	9.73	0.34

**Table 8:** QIDS items are rescaled to the DASS depression scale using 3 calibration items and the Haebara calibration method

item	label	Thr 1	Thr2	Thr3	Mean Thr	Discr
QIDS2	Sleep during the night	−6.24	−2.04	10.65	0.79	0.17
DASS13	I felt sad and depressed	−0.07	1.49	2.51	1.31	2.87
QIDS11	View of myself	0.84	1.62	1.72	1.40	1.59
QIDS5	Feeling sad	−0.04	1.63	2.63	1.40	2.24
DASS26	I felt down-hearted and blue	0.08	1.56	2.60	1.41	3.38
DASS5	I just couldn’t seem to get going	−0.65	1.83	3.65	1.61	1.28
DASS42	I found it difficult to work up the initiative to do things	−0.07	1.73	3.17	1.61	1.72
DASS10	I felt that I had nothing to look forward to	0.55	1.73	2.66	1.65	2.79
DASS17	I felt I wasn’t worth much as a person	0.48	1.82	2.82	1.70	2.44
QIDS10	Concentration/decision making	0.36	1.73	3.06	1.72	1.88
DASS37	I could see nothing in the future to be hopeful about	0.75	1.79	2.64	1.73	3.50
QIDS14	Energy loss	0.15	1.65	3.40	1.73	1.80
DASS34	I felt I was pretty worthless	0.70	1.89	2.76	1.78	2.94
DASS21	I felt that life wasn’t worthwhile	0.74	1.88	2.76	1.79	3.44
DASS38	I felt that life was meaningless	0.88	1.92	2.75	1.85	3.25
DASS24	I couldn’t seem to get any enjoyment out of the things I did	0.47	2.08	3.18	1.91	2.93
DASS31	I was unable to become enthusiastic about anything	0.65	2.10	3.18	1.98	2.57
DASS3	I couldn’t seem to experience any positive feeling at all	0.57	2.17	3.38	2.04	2.72
DASS16	I felt that I had lost interest in just about everything	0.91	2.28	3.31	2.16	2.82
QIDS13	General Interest	0.96	2.47	3.21	2.21	1.81
QIDS16	Feeling restless	1.31	2.62	4.57	2.83	0.98
QIDS15	Feeling slowed down	1.42	2.60	4.49	2.84	1.75
QIDS12	Thoughts of death/suicide	1.35	2.31	4.93	2.86	1.14
QIDS6_7	Appetite change	1.93	3.08	3.81	2.94	1.11
QIDS1	Falling asleep	1.11	3.36	5.38	3.28	0.56
QIDS3	Waking up too early	1.55	3.11	5.38	3.34	0.49
QIDS8_9	Weight change	2.72	3.69	5.79	4.07	0.61
QIDS4	Sleeping too much	3.06	10.40	16.65	10.04	0.32

**Table 9:** DASS depression items are rescaled to the QIDS scale using 3 calibration items and SL rescaling and QIDS items recoded according to the original coding scheme (merge sleep, appetite/weight change and psychomotor items)

item	label	Thr 1	Thr2	Thr3	meanThr	Discr
QIDS1_4^a^	Sleep problems	−3.97	−1.82	2.79	−1.00	0.50
DASS13	I felt sad and depressed	−2.12	0.71	2.55	0.38	1.59
DASS26	I felt down-hearted and blue	−1.85	0.83	2.71	0.56	1.87
DASS5	I just couldn’t seem to get going	−3.17	1.32	4.60	0.92	0.71
DASS42	I found it difficult to work up the initiative to do things	−2.12	1.14	3.74	0.92	0.95
DASS10	I felt that I had nothing to look forward to	−1.00	1.13	2.82	0.99	1.54
DASS17	I felt I wasn’t worth much as a person	−1.13	1.29	3.10	1.09	1.35
DASS37	I could see nothing in the future to be hopeful about	−0.64	1.25	2.79	1.13	1.93
QIDS11	View of myself	0.70	1.34	1.42	1.15	1.99
QIDS5	Feeling sad	−0.02	1.34	2.16	1.16	2.80
DASS34	I felt I was pretty worthless	−0.74	1.43	3.01	1.23	1.62
DASS21	I felt that life wasn’t worthwhile	−0.65	1.40	2.99	1.25	1.90
DASS38	I felt that life was meaningless	−0.40	1.48	2.98	1.35	1.80
QIDS10	Concentration/decision making	0.31	1.44	2.53	1.43	2.29
DASS24	I couldn’t seem to get any enjoyment out of the things I did	−1.15	1.76	3.75	1.45	1.62
QIDS14	Energy loss	0.14	1.38	2.84	1.46	2.13
DASS31	I was unable to become enthusiastic about anything	−0.82	1.81	3.76	1.58	1.42
DASS3	I couldn’t seem to experience any positive feeling at all	−0.97	1.94	4.13	1.70	1.50
QIDS15_16^b^	Psychomotor problems	0.57	1.58	3.03	1.72	1.61
QIDS13	General Interest	0.80	2.04	2.65	1.83	2.22
DASS16	I felt that I had lost interest in just about everything	−0.35	2.12	3.99	1.92	1.56
QIDS6_9^c^	Appetite/Weight change	1.29	2.12	3.27	2.23	0.96
QIDS12	Thoughts of death/suicide	1.12	1.89	4.02	2.34	1.42

^a^Highest of four sleep items

^b^Highest of Psychomotor problems

^c^Highest of appetite/weight change items
